# The matricellular protein CCN5 prevents anti-VEGF drug-induced epithelial-mesenchymal transition of retinal pigment epithelium

**DOI:** 10.1038/s41598-024-63565-z

**Published:** 2024-06-17

**Authors:** Sora Im, Min Ho Song, Muthukumar Elangovan, Kee Min Woo, Woo Jin Park

**Affiliations:** 1 College of Life Sciences, Gwangju Institute of Science and Technology, Gwangju, 61005 Korea; 2 Olives Biotherapeutics, Inc., Gwangju, 61005 Korea

**Keywords:** Macular degeneration, Retinal diseases

## Abstract

Age-related macular degeneration (AMD) is one of the major causes of blindness in the elderly worldwide. Anti-vascular endothelial growth factor (VEGF) drugs have been widely used to treat the neovascular type of AMD (nAMD). However, VEGF acts not only as a pro-angiogenic factor but also as an anti-apoptotic factor in the eyes. In this study, we found that anti-VEGF drugs, including bevacizumab (Bev), ranibizumab (Ran), and aflibercept (Afl), induced epithelial-mesenchymal transition (EMT) in ARPE-19 cells in vitro, accompanied by the induction of CCN2, a potent pro-fibrotic factor. Similarly, intravitreal injection of Afl into mouse eyes resulted in EMT in the retinal pigmented epithelium (RPE). Co-treatment with CCN5, an anti-fibrotic factor that down-regulates CCN2 expression, significantly attenuated the adverse effects of the anti-VEGF drugs both in vitro and in vivo. Inhibition of the VEGF signaling pathway with antagonists of VEGF receptors, SU5416 and ZM323881, induced EMT and up-regulated CCN2 in ARPE-19 cells. Additionally, knock-down of CCN2 with siRNA abolished the adverse effects of the anti-VEGF drugs in ARPE-19 cells. Collectively, these results suggest that anti-VEGF drugs induce EMT in RPE through the induction of CCN2 and that co-treatment with CCN5 attenuates the adverse effects of anti-VEGF drugs in mouse eyes.

## Introduction

Age-related macular degeneration (AMD) is a retinal degenerative disease that leads to irreversible vision loss in individuals over 50 years old in developed countries^1,2^. AMD is classified into two distinctive types: “wet” or neovascular AMD (nAMD) and “dry” or atrophic AMD. nAMD is characterized by the growth of abnormal blood vessels, known as choroidal neovascularization (CNV), into the macula^2^. Extensive studies have revealed that vascular endothelial growth factor (VEGF) plays a major role in the underlying mechanisms of CNV formation^3^. Anti-VEGF therapies have been widely used as a gold standard treatment for nAMD. However, a significant portion of nAMD patients treated with anti-VEGF drugs have reported no benefits or even worsening of visual function, often accompanied by subretinal fibrosis^4–6^.

The retinal pigmented epithelium (RPE) is a highly polarized monolayer structure that demarcates the retinal and choroidal layers and plays a pivotal role in maintaining visual function^5^. Upon various pathological stimuli, RPE cells undergo a cellular process named epithelial-mesenchymal transition (EMT), which results in the loss of epithelial characteristics and the acquisition of mesenchymal characteristics. These cellular and morphological changes of RPE cells are associated with subretinal fibrosis and the related pathological progression observed in eyes with nAMD^7–9^. Therefore, it has been proposed that inhibition or reversal of EMT in RPE cells can be beneficial in patients with nAMD.

CCN5, also known as Wnt-1-induced signaling protein-2 (WISP-2), is a 27 kDa secreted protein and a member of the cell communication network (CCN) family (CCN1 ~ 6)^10^. Unlike other CCN proteins that contain four distinct structural domains, insulin-like growth factor binding protein (IGFBP), von Willebrand factor (VWC), thrombospondin (TSP-1), and cysteine knot (CT), CCN5 lacks the CT domain^11,12^. Due to this structural feature, CCN5 was thought to be a dominant negative regulator of other CCN proteins^13^. Consistent with this proposal, we previously showed that CCN5 prevents cardiac fibrosis partially through the down-regulation of CCN2, a potent pro-fibrotic molecule also known as connective tissue growth factor (CTGF)^14^. Since CCN2 is involved in EMT in various tissues, including RPE, we hypothesized that CCN5 can be beneficial in clinical situations where EMT is associated. We previously demonstrated that CCN5 inhibits and even reverses TGF-β-induced EMT in ARPE-19 cells in vitro^15^, and that it also inhibits CNV and the accompanying EMT of RPE in vivo in a laser-induced murine model^16^.

In the present study, we show that anti-VEGF drugs, including bevacizumab (Bev), ranibizumab (Ran), and aflibercept (Afl), can induce EMT in ARPE-19 cells in vitro and in the RPE of mouse eyes in vivo. We further show that co-treatment with the CCN5 protein ameliorates these adverse effects of anti-VEGF drugs in vitro and in vivo. We also demonstrate that blocking VEGF signaling pathway induces EMT in RPE and that this adverse consequence can be prevented by co-treatment with CCN5.

## Results

### Anti-VEGF drugs induce EMT in ARPE-19 cells

**Figure 1:**
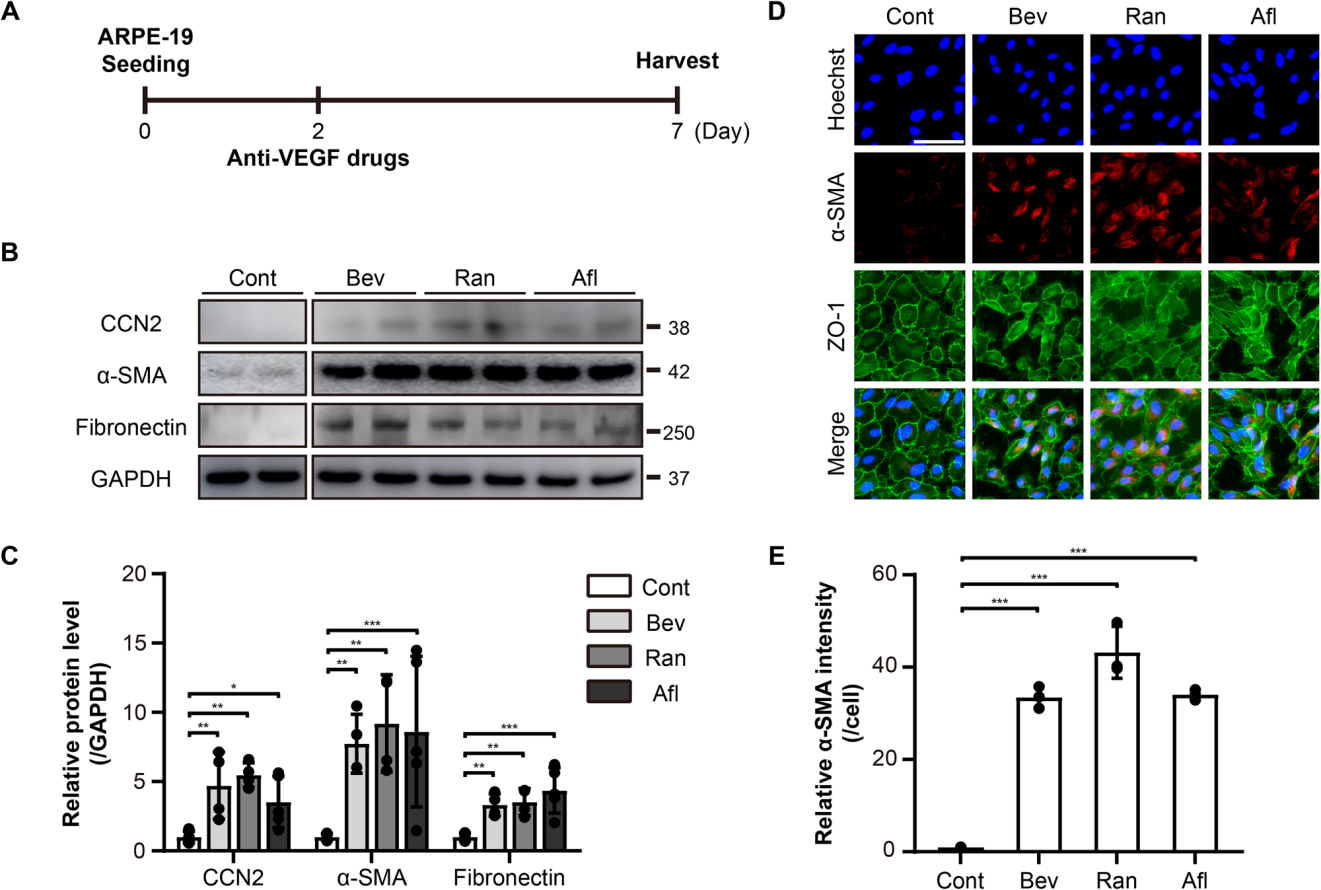
Anti- VEGF drugs induce EMT in ARPE-19 cells. (**A**) An experimental scheme. The ARPE-19 cells were treated with anti-VEGF drugs, bevacizumab (Bev), ranibizumab (Ran), and aflibercept (Afl), at 2× clinical doses. (**B**) Cell lysates from ARPE-19 cells were immunoblotted with antibodies against CCN2, α-smooth muscle actin (α-SMA), fibronectin, and GAPDH. Data are representatives of more than three independent experiments. (**C**) Protein levels were quantified using Image-J software and plotted. Bars show the mean ± SD. (n = 4–6 per group; one-way ANOVA; *p < 0.05, **p < 0.01, ***p < 0.001) (**D**) Immunofluorescence images of ARPE-19 cells stained with anti-α-SMA and anti-zonula occludens-1 (ZO-1) antibodies, and Hoechst 33342. Representative images are shown. Scale bar, 50 μm. (**E**) The intensity of α-SMA per cell was quantified and plotted using Image-J software. Bars show the mean ± SD. (n = 3 per group; one-way ANOVA; ***p < 0.001).

ARPE-19 cells are human RPE cell lines that have been widely used for the study of RPE functions in vitro. The effects of anti-VEGF drugs in ARPE-19 cells have been controversial. Many previous studies have demonstrated that anti-VEGF drugs including Bev, Ran, and Afl have no effects on the viability and proliferation of ARPE-19 cells^17–21^, while other studies have reported that Bev induced EMT in ARPE-19 cells^22–24^. To address this issue, ARPE-19 cells were treated with various doses of Bev, Ran, and Afl for five days, followed by western blotting and immunocytochemistry (Fig. 1). When treated at doses equivalent to clinical doses (Bev, 0.312 mg/mL; Ran, 0.125 mg/mL; Afl, 0.5 mg/mL) all three anti-VEGF drugs had slight but inconsistent effects on ARPE-19 cells. However, when treated at doses equivalent to twice the clinical doses, all anti-VEGF drugs consistently induced the expression of EMT marker proteins, α-SMA and fibronectin, as well as a potent pro-fibrotic molecule, CCN2 within five days (Fig. 1B, C). Immunocytochemistry also showed a significant elevation in the expression of α-SMA with all anti-VEGF drug treatments (Fig. 1D, E). ZO-1, a tight junction protein, was used to demarcate individual cells (Fig. 1D). These drugs did not affect cell viability (data not shown). Altogether, these data suggest that all the tested anti-VEGF drugs have the potential to induce EMT in ARPE-19 cells.

### Recombinant CCN5 protein inhibits EMT induced by anti-VEGF drugs in ARPE-19 cells

**Figure 2:**
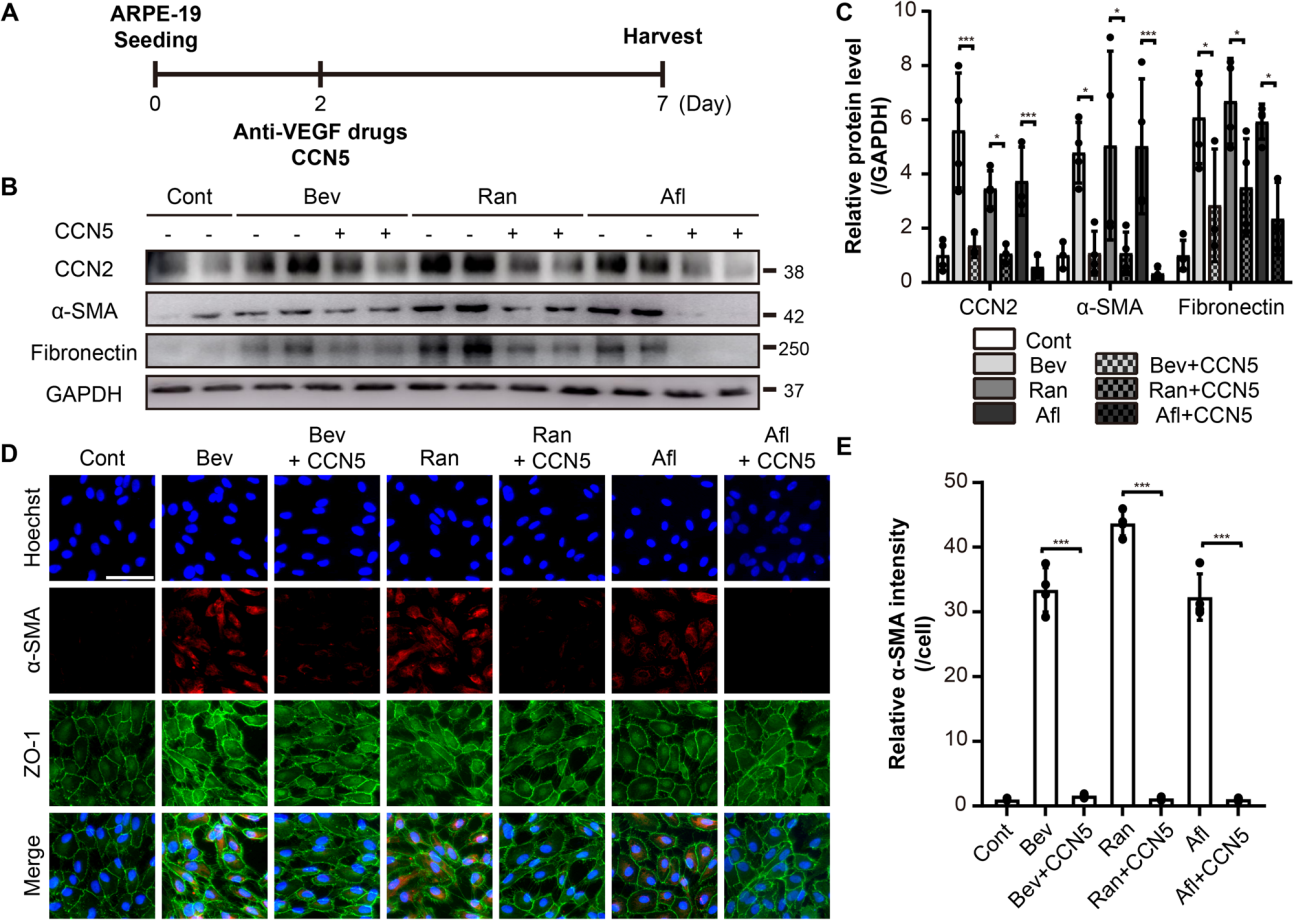
Recombinant CCN5 protein inhibits EMT induced by anti-VEGF drugs in ARPE-19 cells. (**A**) An experimental scheme. The ARPE-19 cells treated with anti-VEGF drugs, Bev, Ran, and Afl, at 2× clinical doses in the absence or presence of recombinant CCN5 protein (500 ng/mL). (**B**) Cell lysates from ARPE-19 cells were immunoblotted with antibodies against CCN2, α-SMA, fibronectin, and GAPDH. Data are representatives of more than three independent experiments. (**C**) Protein levels were quantified using Image-J software and plotted. Bars show the mean ± SD. (n = 4 per group; one-way ANOVA; *p < 0.05, ***p < 0.001) (**D**) Immunofluorescence images of ARPE-19 cells stained with anti-α-SMA and anti-ZO-1 antibodies, and Hoechst 33342. Representative images are shown. Scale bar, 50 μm. (**E**) The intensity of α-SMA per cell was quantified and plotted using Image-J software. Bar show the mean ± SD. (n = 3–4 per group; one-way ANOVA; ***p < 0.001).

We previously showed that CCN5 inhibits TGF-β-induced EMT in ARPE-19 cells^15^. To examine whether CCN5 can also inhibit EMT induced by the anti-VEGF drugs, ARPE-19 cells were treated with Bev, Ran, and Afl in the absence or presence of the recombinant CCN5 protein (500 ng/mL) for five days (Fig. 2A). Consistent with our previous findings, CCN5 down-regulated the expression of CCN2 induced by anti-VEGF drugs. Moreover, the expression levels of α-SMA and fibronectin elevated by anti-VEGF drugs were normalized by CCN5 co-treatment, as shown by western blotting (Fig. 2B, C). Immunocytochemistry confirmed a significant reduction in α-SMA expression induced by anti-VEGF drugs upon CCN5 co-treatment (Fig. 2D, E). Taken together, these results indicate that CCN5 inhibits EMT induced by the anti-VEGF drugs. It is of note that CCN5 alone did not affect the expression of the EMT markers (Supplementary Fig. 1).

### Recombinant CCN5 protein inhibits EMT induced by Afl in mouse eyes

**Figure 3:**
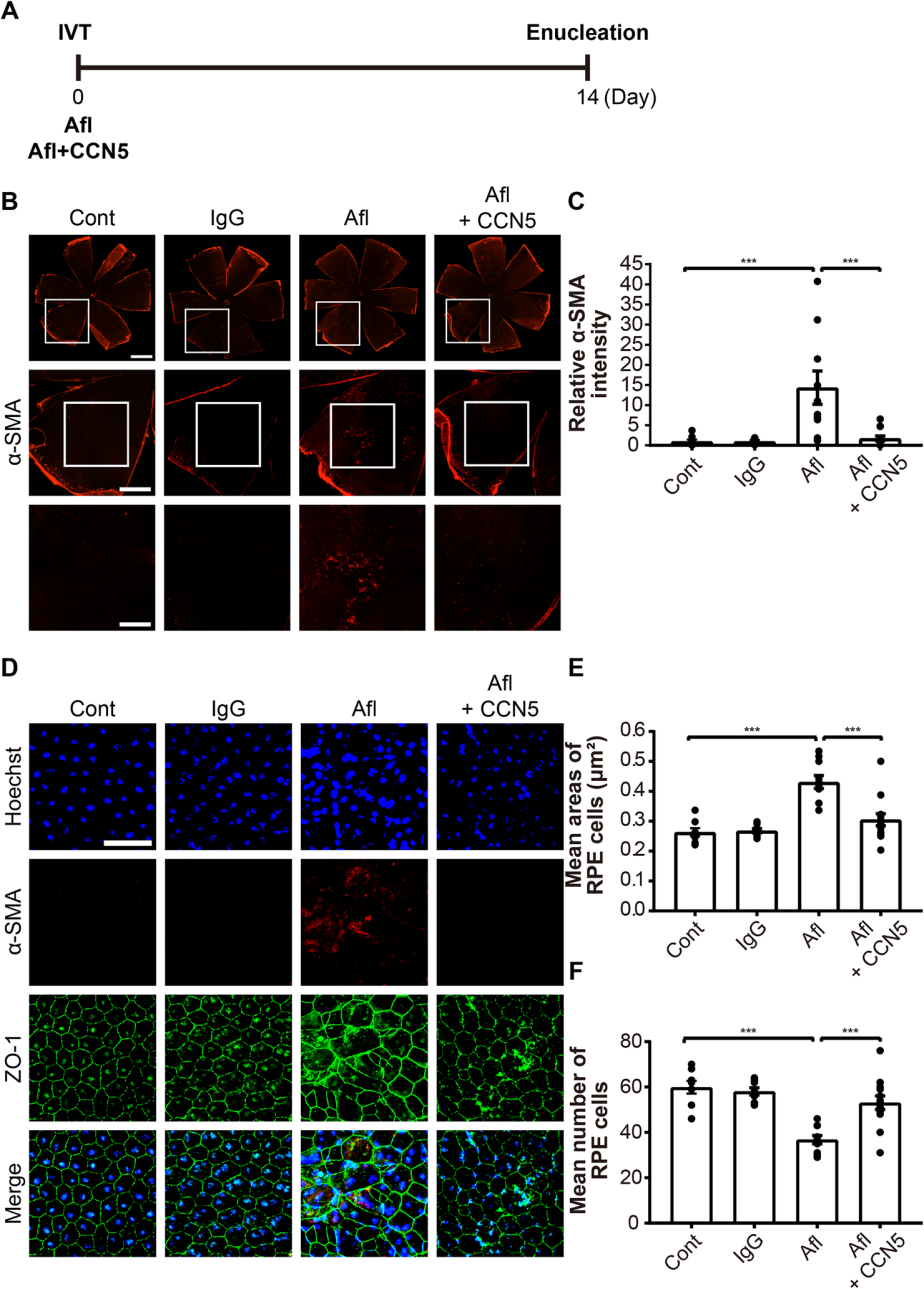
Recombinant CCN5 protein inhibits EMT induced by aflibercept (Afl) in mouse eyes. (**A**) An experimental scheme. Normal human IgG (40 mg/eye), Afl (40 μg/eye), and Afl and CCN5 (40 ng/eye) were intravitreally injected. Mice were sacrificed, and eyes were enucleated at 14 days post-injection. (**B**) Flat mounts of RPE/choroid complex were stained with anti-α-SMA antibody. Representative images are shown. Scale bar, 1000, 500, and 250 μm. (**C**) The intensity of α-SMA positive areas was quantified by Image-J software and plotted. (**D**) Flat mounts of RPE/choroid complex were stained with anti-α-SMA and anti-ZO-1 antibodies, and Hoechst 33342. Representative images are shown. Scale bar, 50 μm. (**E**) The mean number of RPE cells in 100 × 100 μm area was manually counted and plotted. (**F**) The mean size of RPE cells in 100 × 100 μm area was manually calculated and plotted. Error bars = SEM. (n = 8, Cont; n = 8, IgG; n = 10, Afl; n = 13, Afl+CCN5; one-way ANOVA; ***p < 0.001).

We then examined the effects of Afl and CCN5 in vivo in mouse eyes. Bev and Ran were not tested in this experiment because neither of them possesses considerable affinities to mouse VEGF^25–27^. Afl was intravitreally injected into mouse eyes at a dose of 40 μg/eye. This dose is the one routinely used in other studies where the anti-neovascularization activity of Afl was evaluated in mice^28–30^. Afl was injected alone or in combination with recombinant CCN5 protein (40 ng/eye). The eyes were enucleated and the RPE/choroid complex was separated at 14 days post-injection (Fig. 3A). Immunohistochemistry of the flat-mounted RPE-choroid complex exhibited that the α-SMA expression was significantly elevated by Afl, which was significantly inhibited by the co-treatment with CCN5 (Fig. 3B). This finding was clearly illustrated by quantitation of the α-SMA-positive areas (Fig. 3C). Immuno-staining with anti-ZO-1 antibody revealed that RPE cells became irregular and enlarged upon the treatment with Afl, and that this abnormality was significantly normalized by CCN5 (Fig. 3D). Mean cell numbers were significantly reduced by Afl, and this abnormality was prevented by the co-treatment with CCN5 (Fig. 3E). Simiarly, mean cell size was significantly increased by Afl, which was prevented by the co-treatment with CCN5 (Fig. 3F). These results indicate that the anti-VEGF drug, Afl, induces EMT accompanied by morphological changes of RPE, and that CCN5 can inhibit the fibrotic deformation induced by Afl.

### Antagonists of VEGF receptors induce EMT and upregulate CCN2 expression in ARPE-19 cells

**Figure 4:**
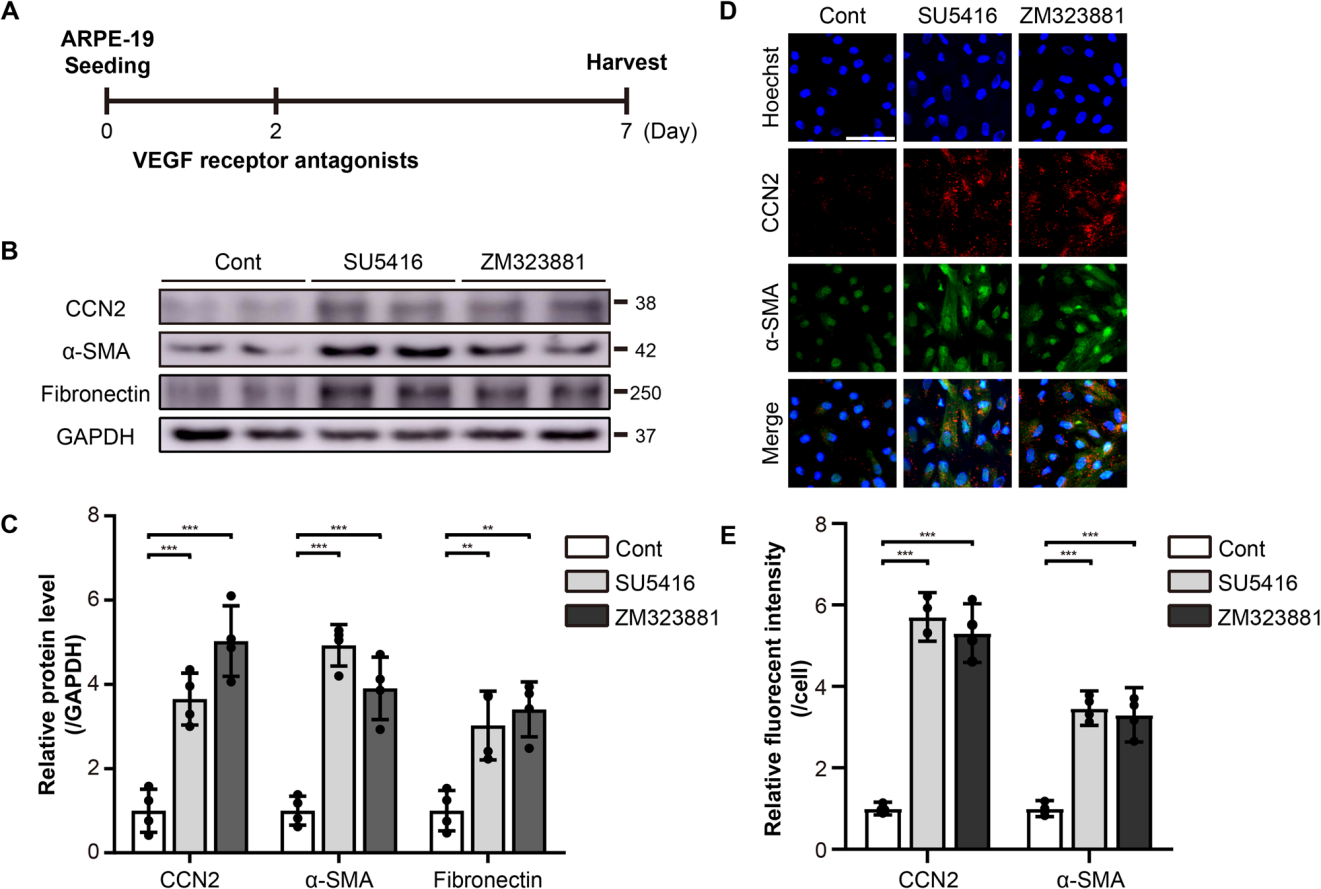
Antagonists of VEGF receptors induce EMT in ARPE-19 cells. (**A**) An experimental scheme. The ARPE-19 cells were treated with 5 μM of SU5416, an antagonist of VEGFR-1/2, or 10 nM of ZM323881, an antagonist of VEGFR-2. (**B**) Cell lysates from ARPE-19 cells were immunoblotted with antibodies against CCN2, α-SMA, fibronectin, and GAPDH. Data are representatives of more than three independent experiments. (**C**) Protein levels were quantified using Image-J software and plotted. Bars show the mean ± SD. (n = 4 per group; one-way ANOVA; **p < 0.01, ***p < 0.001) (**D**) Immunofluorescence images of ARPE-19 cells stained with anti-α-SMA and anti-CCN2 antibodies, and Hoechst 33342. Representative images are shown. Scale bar, 50 μm. (**E**) The intensity of CCN2 or α-SMA per cell was quantified and plotted using Image-J software. Bars show the mean ± SD. (n = 4 per group; one-way ANOVA; ***p < 0.001).

We further pursued to elucidate the mechanism underlying the anti-VEGF drug-induced EMT in ARPE-19 cells. The VEGF signaling pathway is inherently active in ARPE-19 cells at baselines and is suject to further activation (Supplementary Figs. 2 and 3). ARPE-19 cells were treated with 5 μM of SU5416, an antagonist of VEGFR-1/2, or 10 nM of ZM323881, an antagonist of VEGFR-2 for 5 days (Fig. 4A). Western blotting demonstrated that CCN2 and EMT marker proteins, α-SMA and fibronectin, were up-regulated by the treatment with both VEGF receptor antagonists (Fig. 4B, C). Immunocytochemistry also showed an increase in CCN2- and α-SMA-positive cells upon treatment with SU5416 and ZM323881 (Fig. 4D, E). These results indicate that the inhibition of VEGF signaling pathways can induce EMT in ARPE-19 cells, which is correlated with the induction of CCN2 expression.

### Knockdown of CCN2 inhibits EMT induced by Afl in ARPE-19 cells

**Figure 5:**
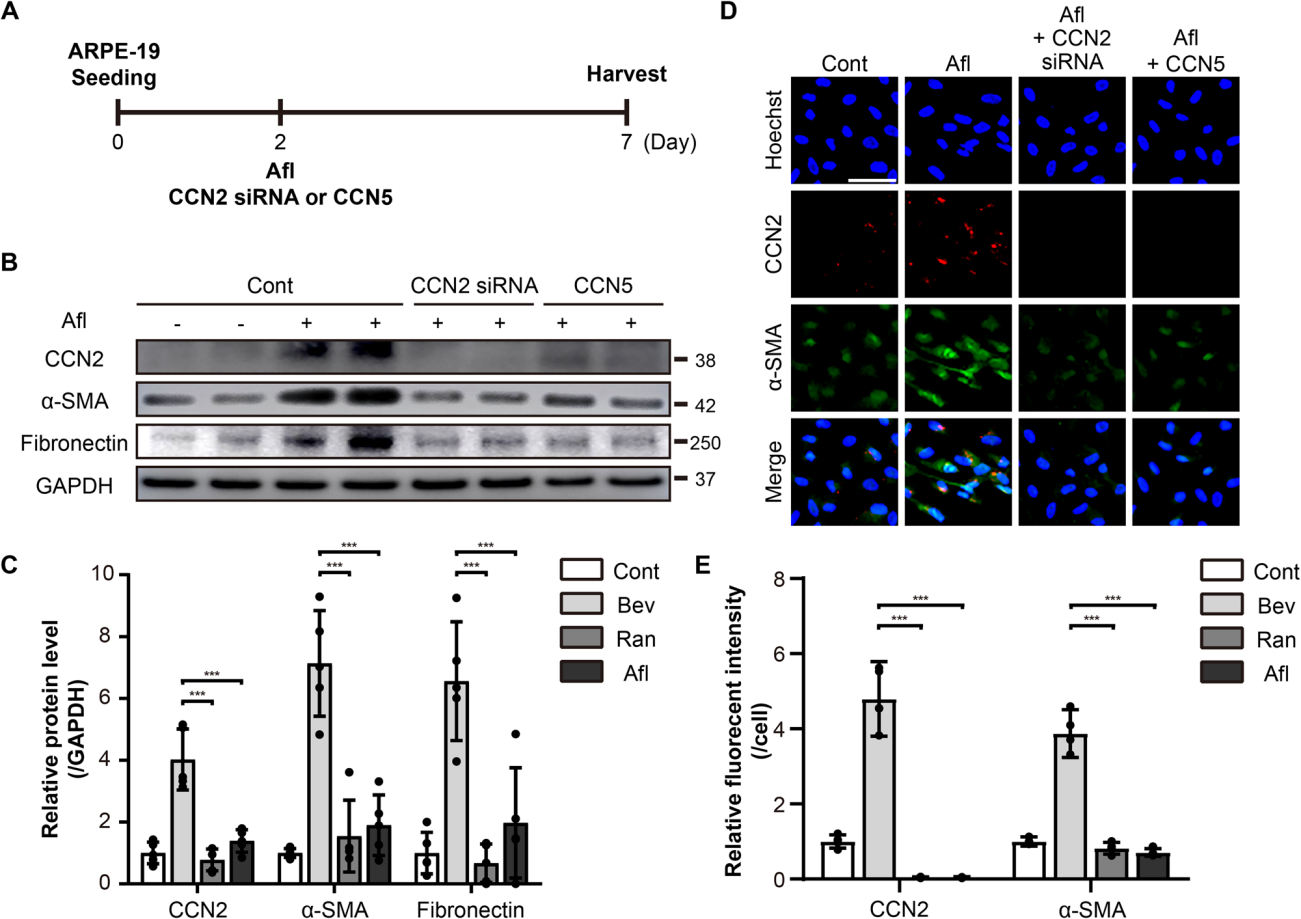
Afl-induced EMT is ameliorated by CCN2 siRNA in ARPE-19 cells. (**A**) An experimental scheme. The ARPE-19 cells were treated with CCN2 siRNA (1 μM) or CCN5 (500 ng/mL) in the presence of 2× Afl for 5 days. (**B**) Cell lysates from ARPE-19 cells were immunoblotted with antibodies against CCN2, α-SMA, fibronectin, and GAPDH. Data are representatives of more than three independent experiments. (**C**) Protein levels were quantified using Image-J software and plotted. Bars show the mean ± SD. (n = 5 per group; one-way ANOVA; ***p < 0.001) (**D**) Immunofluorescence images of ARPE-19 cells were stained with anti- α-SMA and anti-CCN2 antibodies, and Hoechst 33342. Representative images are shown. Scale bar, 50 μm. (**E**) The intensity of CCN2 or α-SMA per cell was quantified and plotted using Image-J software. Bars show the mean ± SD. (n = 4 per group; one-way ANOVA; ***p < 0.001).

The role of CCN2 in retinal fibrosis has previously been shown^31^. To test whether CCN2 is also involved in the anti-VEGF drug-induced EMT, ARPE-19 cells were treated with CCN2 siRNA in combination with Afl for five days (Fig. 5A). CCN2 siRNA completely blocked the induction of CCN2 by Afl. Furthermore, the Afl-mediated induction of α-SMA and fibronectin was significantly ameliorated by CCN2 siRNA, as shown by western blotting (Fig. 5B, C). Immunocytochemistry confirmed that CCN2 siRNA inhibited the induction of α-SMA by Afl in ARPE-19 cells (Fig. 5D, E). The effects of CCN2 siRNA were comparable to those of CCN5 in blocking the Afl-mediated induction of EMT (Fig. 5B–E). These results suggest that Afl, and most likely other anti-VEGF drugs, induces EMT by activating CCN2 signaling pathways, and that CCN5 inhibits anti-VEGF drug-induced EMT through the downregulation of CCN2 expression.

## Discussion

VEGF is well known for its activity as a pro-angiogenic factor^32,33^. Thus, anti-VEGF therapies exhibit clear benefits for patients with nAMD. Besides the pro-angiogenic activity, VEGF displays protective effects in diverse tissues including neuronal cells. For example, VEGF protects hippocampal neurons from hypoxic damages^34^. In the eyes, VEGF was shown to act as an anti-apoptotic factor for photoreceptors^35^, Müller cells^36^, and RPE^37^. Considering the protective role of VEGF in the eyes, it is conceivable to observe that anti-VEGF therapies targeted for nAMD are often associated with accelerated deterioration of nAMD to geographic atrophy^38,39^. Another pressing issue is that a substantial portion of patients with nAMD do not respond to anti-VEGF therapies^40,41^. These findings necessitate the need for effective yet safer modalities for the treatment of nAMD.

In this study, we demonstrated that anti-VEGF drugs, including Bev, Ran, and Afl, induced EMT in ARPE-19 cells when treated at 2× clinical doses (Fig. 1). Clinical doses used in this in vitro study were deduced from the dosage of drugs administered in clinics and the average volume of human vitreous humor. No adverse effects of anti-VEGF drugs were reported in ARPE-19 cells in a number of previous studies. For example, Bev did not affect the viability of ARPE-19 cells when treated at 2.0 mg/mL for 24 h^20^. Ran and Afl were not cytotoxic to ARPE-19 cells when treated at 1.0 mg/mL for 24 h^19^. Bev, Ran, and Afl were not cytotoxic to ARPE-19 cells when treated at 0.3125 mg/mL, 0.125 mg/mL, and 2 mg/mL, respectively, for 72 h^18^. Bev, Ran, and Afl did not affect the viability and mitochondrial membrane potential of ARPE-19 cells when treated at the clinical doses for 24 h^17^. In contrast, adverse effects of these drugs were reported in several other studies. For example, Bev induced EMT in ARPE-19 cells when treated at 1.25 mg/mL for 24 h^22^. Similarly, Bev induced EMT in ARPE-19 cells by regulating Notch signaling when treated at 0.25 mg/mL for 24 h^42^. Bev affected the proliferation, phagocytosis, and membrane potential of ARPE-19 cells when treated at 0.25 mg/mL for 48 h^23^. Bev induced EMT in ARPE-19 cells by regulating the TGF-β1-SMAD2/3 signaling pathway when treated at 0.25 mg/mL for 48 h. Collectively, it appears that anti-VEGF drugs do not affect cell viability but do induce EMT and affect cell function when treated at clinical or higher doses in ARPE-19 cells.

Consistent with this hypothesis, we did not observe any effects of the anti-VEGF drugs on cell viability at both clinical and 2× clinical doses but observed pro-EMT effects of the drugs at 2× clinical doses (Fig. 1). We further showed that blocking VEGF signaling by antagonists of VEGF receptors led to EMT in ARPE-19 cells (Fig. 4). Therefore, it appears that the adverse effects of anti-VEGF drugs shown in this and other previous studies might be due to a reduction of VEGF signaling below a hypothetical threshold by the drugs.

CCN2, also known as CTGF, acts as a pro-fibrotic factor in diverse pathological situations^43–45^. It was shown that Bev up-regulated CCN2 expression in ARPE-19 cells via Fc-FcR interaction^22^. In patients with proliferative diabetic retinopathy (PDR), CCN2 correlated positively, and VEGF negatively, with the degree of fibrosis^46^. Immunofluorescence microscopy revealed that signals for TGF-β2 and CCN2 were significantly intensified in PDR patients who received Bev therapy compared with PDR patients who did not receive Bev therapy^47^. In consistent with these studies, we observed that CCN2 is induced by the anti-VEGF drugs (Figs. 1, 2). We further found that the knock-down of CCN2 abolished the adverse effects of the anti-VEGF drugs in ARPE-19 cells (Fig. 5), which indicates that CCN2 is the key factor mediating the adverse effects, not just a bystander.

CCN5 is an anti-fibrotic factor in various tissues^13,48^. We previously showed that CCN5 is secreted from source cells via its amino-terminal signal sequence and is subsequently endocytosed by neighboring target cells, after which it enters the nucleus and functions as a transcriptional co-activator or co-repressor. We also showed that CCN5 directly regulates the expression of SMAD7 at the transcriptional level^49^, and Sabbah et al. showed that CCN5 functions as a transcriptional repressor of TGF-β receptor II^50^. CCN5 inhibits TGF-β-induced EMT in ARPE-19 cells^15^. More surprisingly, CCN5 reverses pre-formed EMT in ARPE-19 cells, as it was previously shown to reverses pre-formed fibrosis in hearts^14^.

Based on the results of this study, we propose that anti-VEGF drugs can be safer when it is administered in combination with CCN5. This study lays a foundation for the development of an efficient yet safer therapeutic modality for nAMD.

## Methods

### Cells and cell culture

Human RPE cell line (ARPE-19) was purchased from the American type culture collection (ATCC, Manassas, VA). The cells were cultured and maintained in DMEM/F-12 (Welgene, Gyeongsan, Korea) supplemented with 10% fetal bovine serum (HyClone, Logan, UT) and 1% penicillin–streptomycin (Gibco, Gaithersburg, MD). The cells were incubated at 37 °C and 5% CO_2_ until they reached 90% confluency for this study.

### Anti-VEGF drugs

Anti-VEGF drugs were treated to ARPE-19 cells for 5 days. Clinical doses used in this in vitro study were: Bev (Roche, Basel, Switzerland), 0.312 mg/mL; Ran (Novartis, Basel, Switzerland), 0.125 mg/mL; Afl (Bayer, Leverkusen, Germany), 0.5 mg/mL. Afl was also intravitreally injected into mouse eyes (40 μg/eye).

### Recombinant CCN5 protein

Recombinant CCN5 protein was purified as previous described^51^. The CCN5 proteins was treated at a concentration of 500 ng/mL in ARPE-19 cells or intravitreally injected into mouse eyes (40 ng/eye).

### VEGF receptor antagonists

SU5416, an antagonist of VEGFR-1/2, and ZM323881, an antagonist of VEGFR-2, were purchased from Cayman (Ann Arbor, MI). They were dissolved in dimethyl sulfoxide (DMSO; Sigma–Aldrich, St. Louis, MO) and diluted in culture media immediately before treatment. SU5416 and ZM323881 were treated at concentrations of 5 μM and 10 nM, respectively, for 5 days.

To activate the VEGF signaling pathway, VEGF-A (R&D systems, Minneapolis, MN) was treated at concentration of 20 ng/mL for 20 min.

### si RNA

An Accell SMARTpool small interfering RNA (siRNA) targeting human CCN2 (CCN2 siRNA) was purchased from Dharmacon (Lafayette, CO). ARPE-19 cells were transfected with 1 μM of CCN2 siRNA in Accell delivery media according to the manufacturer’s instructions for 5 days.

### Western blotting

ARPE-19 cells were washed with phosphate-buffered saline (PBS) and scrapped. RIPA lysis buffer (1% NP-40, 50 mM Tris–HCl [pH7.4], 150 mM NaCl, and 10 mM NaF) containing protease inhibitor cocktail (PIC; Roche, Basel, Switzerland) was used to extract proteins. The protein concentration was quantified using the Pierce BCA protein assay kit (Thermo Fisher Scientific, Waltham, MA). An equal amount of protein samples was mixed with NuPAGE LDS sample buffer (Invitrogen, Carlsbad, CA), separated by SDS–PAGE, and transferred to polyvinylidene difluoride membrane (PVDF; Merck, Kenilworth, NJ). The membrane was blocked with 5% (w/v) bovine serum albumin (BSA) in Tris-buffered saline containing 0.1% (v/v) Tween 20 (TBS-T) for 1 h at room temperature (RT) and incubated with primary antibodies in 3% (w/v) BSA in TBS-T overnight at 4 °C. They were washed with TBS-T and incubated with appropriate secondary antibodies conjugated to horseradish peroxidase (HRP) in TBS-T for 1 h at RT. After blots were washed, immune complex was developed using an EZ-Western Lumi Pico kit (Dogenbio, Seoul, Korea) and Amersham IamgeQuant 800 (Cytiva, Marlborough, MA). Primary antibodies were anti-CCN2 (Santa Cruz, Dallas, TX), anti-α-smooth muscle actin (α-SMA; Sigma–Aldrich, St. Louis, MO), anti-fibronectin (Abcam, Cambridge, UK), and anti-GAPDH (laboratory made). Secondary antibodies against mouse and rabbit IgG were obtained from Invitrogen (Carlsbad, CA).

### Immunocytochemistry

ARPE-19 cells were cultured in coverslips in 12-well culture plate washed with PBS and fixed using 4% (v/v) paraformaldehyde for 15 min at RT. The cells were permeabilized with 0.5% (v/v) Triton X-100 for 15 min at RT and blocked with 5% (w/v) BSA in PBS for 1 h at RT. The blocked cells were incubated with primary antibodies overnight at 4 °C and washed with PBS. They were then incubated with appropriated secondary antibodies and Hoechst 33342 (Invitrogen, Carlsbad, CA) for 1 h at RT and washed with PBS. They were then mounted on slide glass using a mounting medium (Dako, Santa Clara, CA), and observed under a Zeiss fluorescence microscopy (Zeiss, Oberkochen, Germany). An additional primary antibody is anti-zonula occludens-1 (ZO-1; Invitrogen, Carlsbad, CA). Secondary antibodies labelled with Alexa Flour 488 or 555 against mouse and rabbit were obtained from Invitrogen (Carlsbad, CA).

### Animals and treatment

All animal experiments were conducted in accordance with relevant guidelines and regulations for laboratory animals approved by the Institutional Animal Care and Use Committees of Gwangju Institute of Science and Technology. All efforts were made to minimize animal suffering used for this study. This study is reported in accordance with ARRIVE guidelines. Male C57BL/6 mice were purchased from DBL Inc. (Eumseong, Korea). All mice were housed in equipped animal facility with temperature of 18–23 °C and humidity at 40–60%, under 12/12 h light/dark cycle, and had free access to food and water. Intravitreal injection was performed under anesthesia induced by intraperitoneal injection of a mixture of 80 mg/kg zoletil 50 (Virbac, Carros Cedex, France) and 20 mg/kg Rompun (Bayer, Leverkusen, Germany) and pupil dilation induced by a mixture of 0.5% (w/v) tropicamid and 0.5% (w/v) phenylephrin (Hanmi Pharm, Seoul, Korea). 40 μg/eye of human IgG (R&D system, Minneapolis, NE), 40 μg/eye of Afl, 40 ng/eye of the CCN5 protein were intravitreally injected using a microliter syringe with 33-G needle (Hamilton, Reno, NV).

### Flat mounting and immunohistochemistry

Mice were sacrificed with a CO_2_ chamber under deep anesthesia as described above. The eyes were enucleated and fixed with 4% (v/v) paraformaldehyde for 30 min at RT. Cornea, lens, and retina were removed from RPE/choroid complex, and they were washed with PBS. RPE/choroid complex were incubated with primary antibodies in PBlec solution (0.1 mM CaCl_2_⋅2H_2_O, 0.1 mM MgCl_2_, 0.1 mM MnCl2⋅4H_2_O, and 1% (v/v) Triton X-100, pH 6.8) overnight at 4 °C. They were washed with PBS and then incubated with secondary antibodies and Hoechst 33342 in PBS for 2 h at RT. After washing with PBS, RPE/choroid complex were then mounted on slide glass using a mounting medium, and observed under an Olympus research slide scanner (Olympus, Tokyo, Japan), and Olympus confocal microscopy (Olympus, Tokyo, Japan).

### Image analysis and cell counting

Immunofluorescence images were randomly selected from each mouse eye. Image-J software (National Institute of Health, Bethesda, MD) was used to estimate the α-SMA-positive area of immunofluorescence images. Images were imported to Image-J software, inverted, and converted to an 8-bit image. The threshold was equally adjusted and analyzed by the “analyze particles” tool to calculate % area. Image-J software was also used to estimate the intensity of fluorescein of immunofluorescence images. The images were cropped to 2500 μm^2^, imported to Image-J software, and inverted. The images were converted to 8-bit and the mean intensity was measured. Immunofluorescence images of ZO-1 were used for RPE cell counting. The number of RPE cells in 2500 μm^2^ were manually counted, and the area was divided by the number of RPE cells to calculate the mean areas of individual RPE cells.

### Statistical analysis

All experiments were repeated independently at least three times. Statistical significance of difference was analyzed by one-way analysis of variance (ANOVA) using GraphPad Prism 7 software (GraphPad Software, San Diego, CA). A p-value less than 0.05 was considered to indicate statistical significance. Each bar represents the SEM for parametric data.

### Supplementary Information

Supplementary Figure 1.

Supplementary Information.

## Data Availability

All relevant data are within the paper and its Supporting Information files.
