# Supplementary material for: The matricellular protein CCN5 prevents anti-VEGF drug-induced epithelial-mesenchymal transition of retinal pigment epithelium
Source: Sci Rep. 2024 Jun 17;14:13920. doi: 10.1038/s41598-024-63565-z (PMC11183261; doi:10.1038/s41598-024-63565-z)
Supplement: Supplementary file 1 — Supplementary Figure 1. [file 41598_2024_63565_MOESM1_ESM.docx]

**Supplementary information Western blot row image**

**The matricellular protein CCN5 prevents anti-VEGF drug-induced epithelial-mesenchymal transition of retinal pigment epithelium**

Sora Im ^1^, Min Ho Song ^2^, Muthukumar Elangovan ^1^, Kee Min Woo ^1^ and Woo Jin Park ^1,2*^

Figure 1b


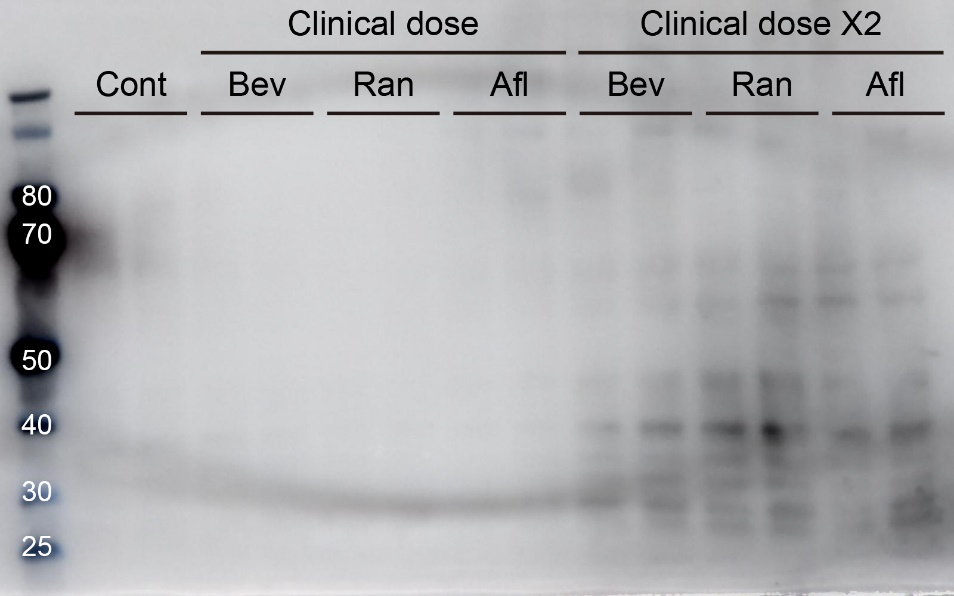


CCN2 (38-kDa)


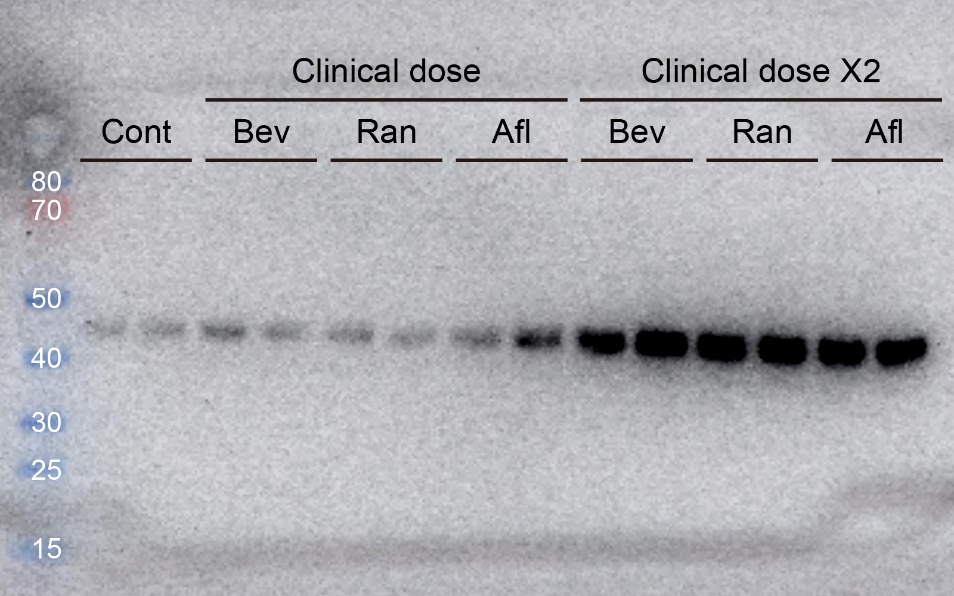


α-SMA (42-kDa)


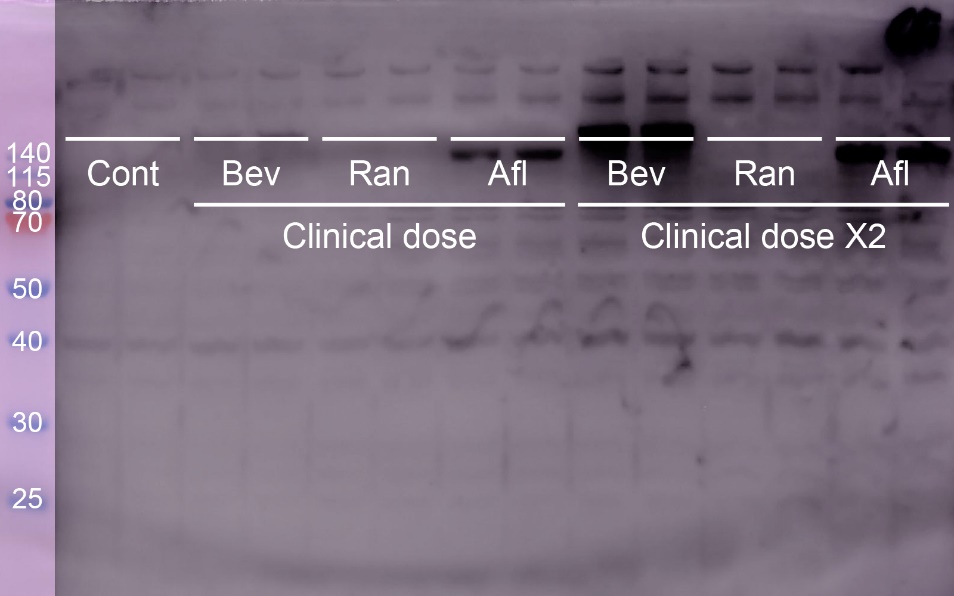


Fibronectin (250-kDa)


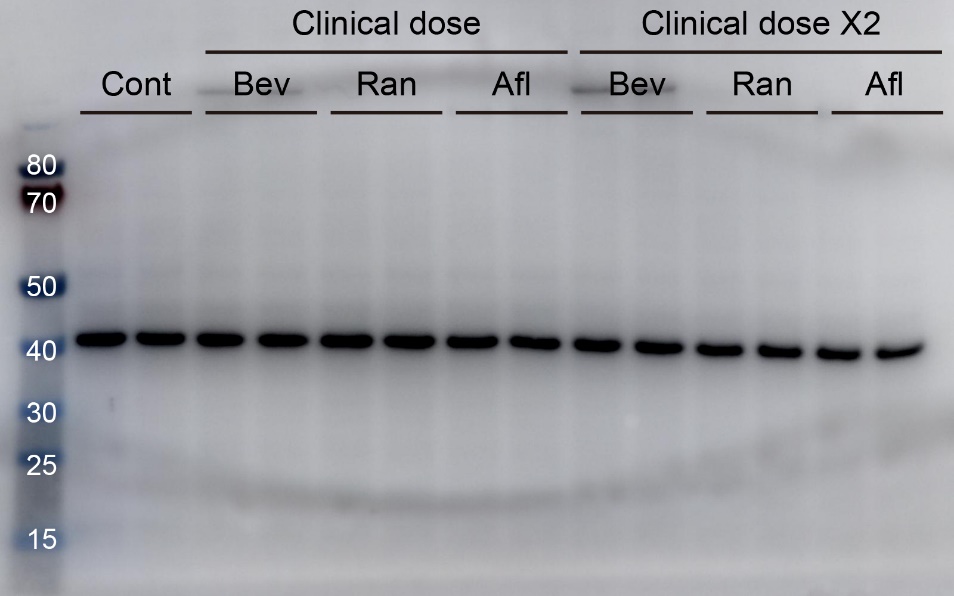


GAPDH (37-kDa)

Figure 2b


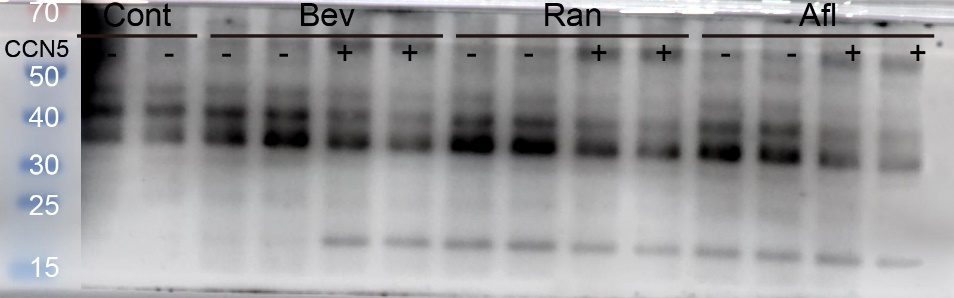


CCN2 (38-kDa)


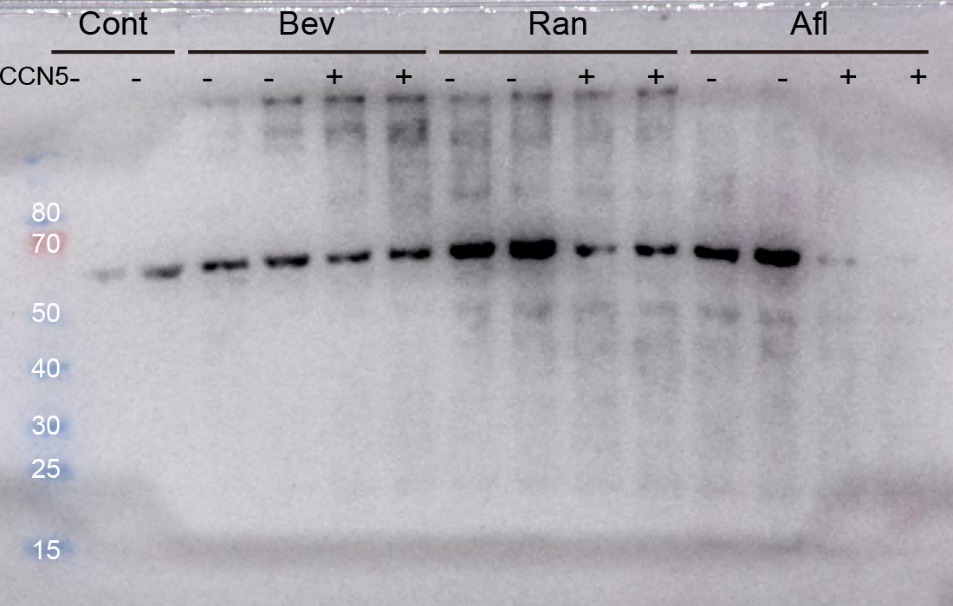


α-SMA (42-kDa)


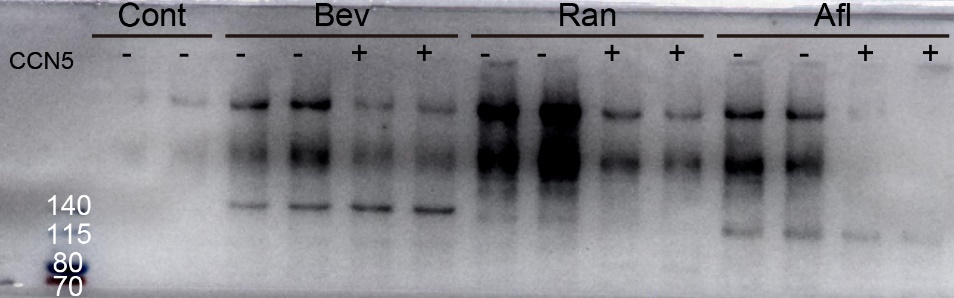


Fibronectin (250-kDa)


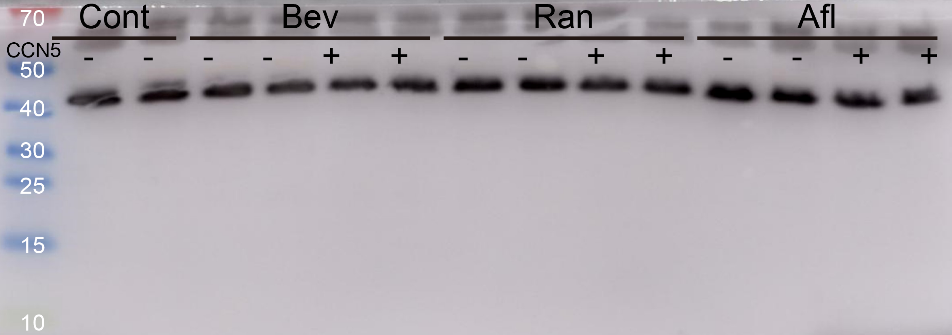


GAPDH (37-kDa)

Figure 4b


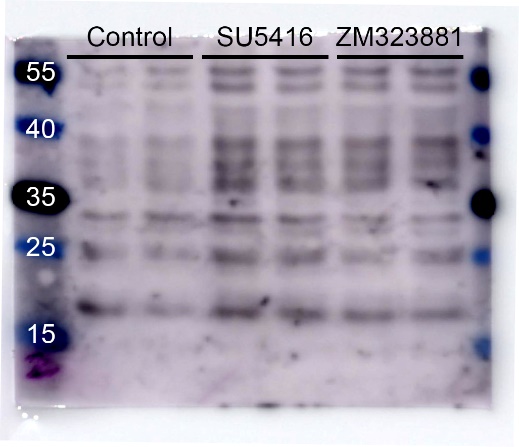


CCN2 (38-kDa)


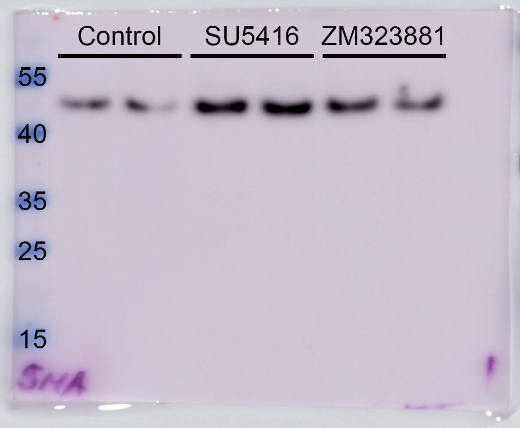


α-SMA (42-kDa)


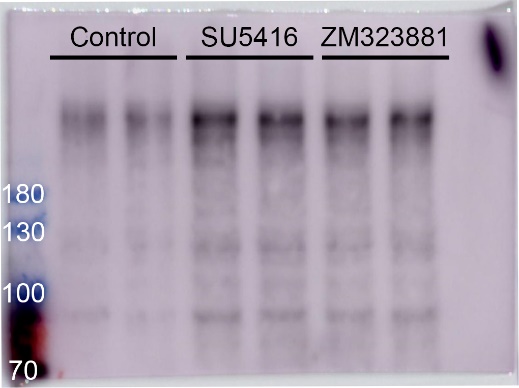


Fibronectin (250-kDa)


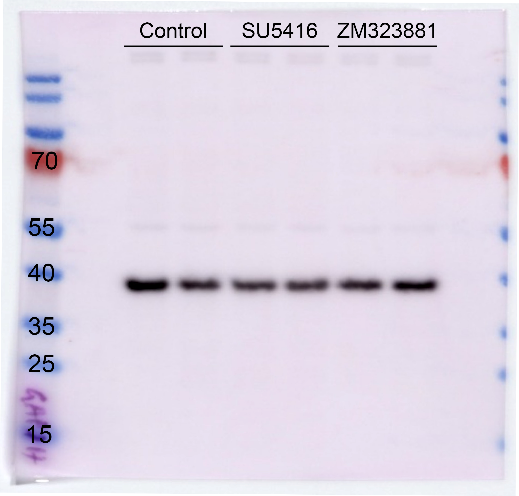


GAPDH (37-kDa)

Figure 5b

CCN2 (38-kDa)


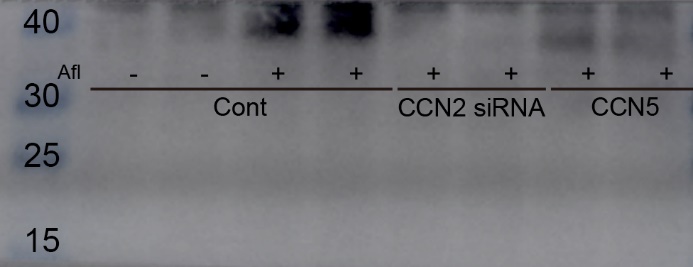


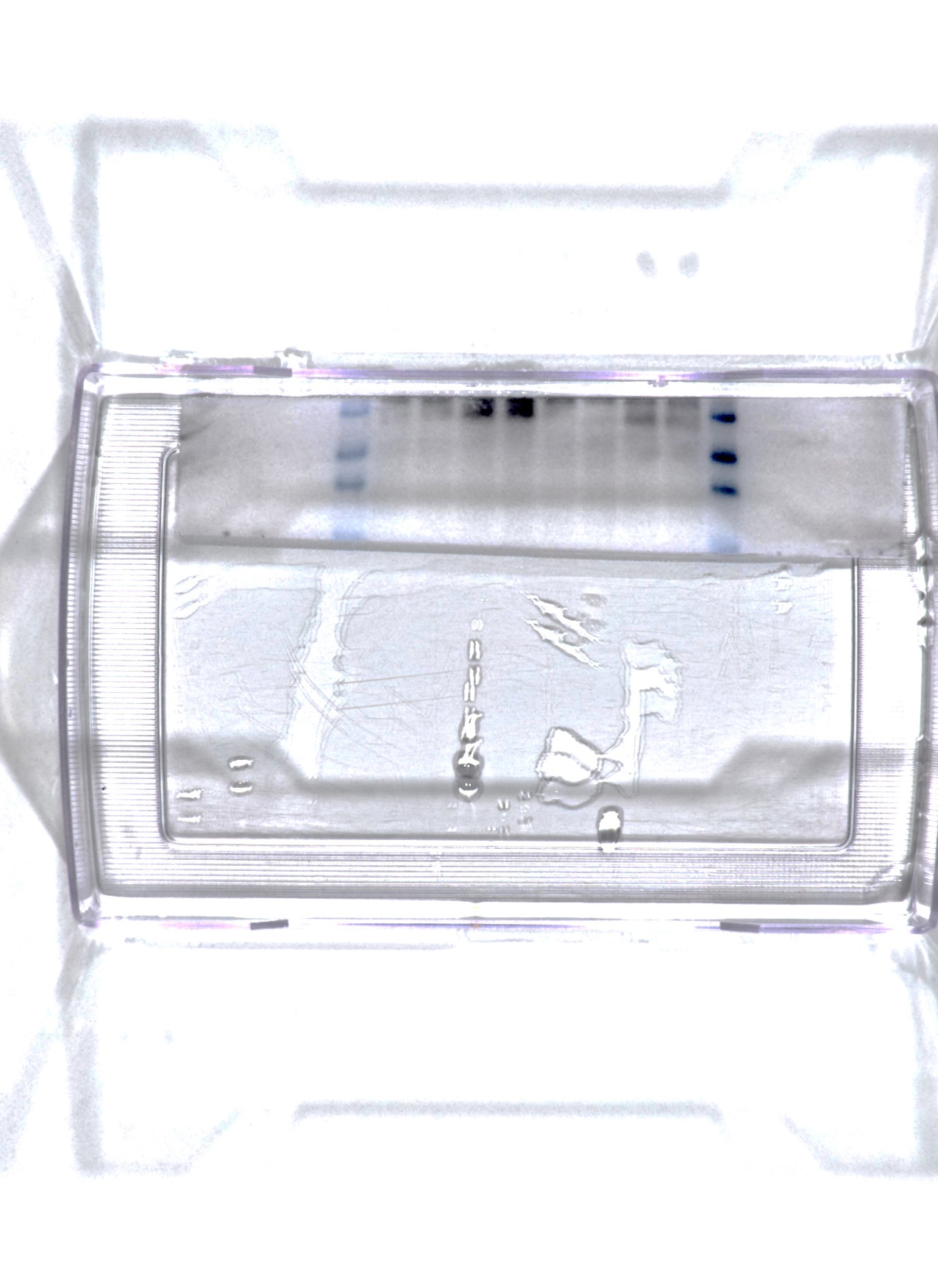
 CCN2 original blot


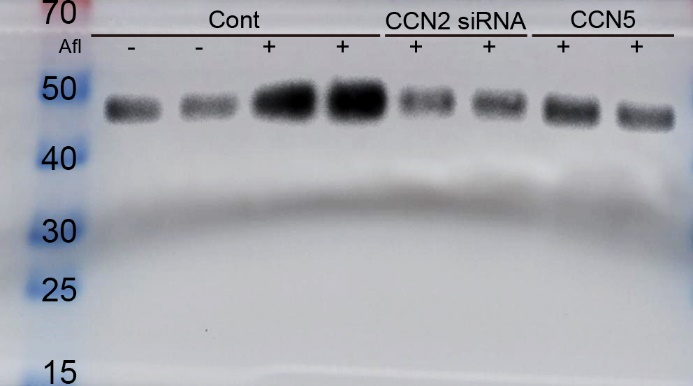


α-SMA (42-kDa)


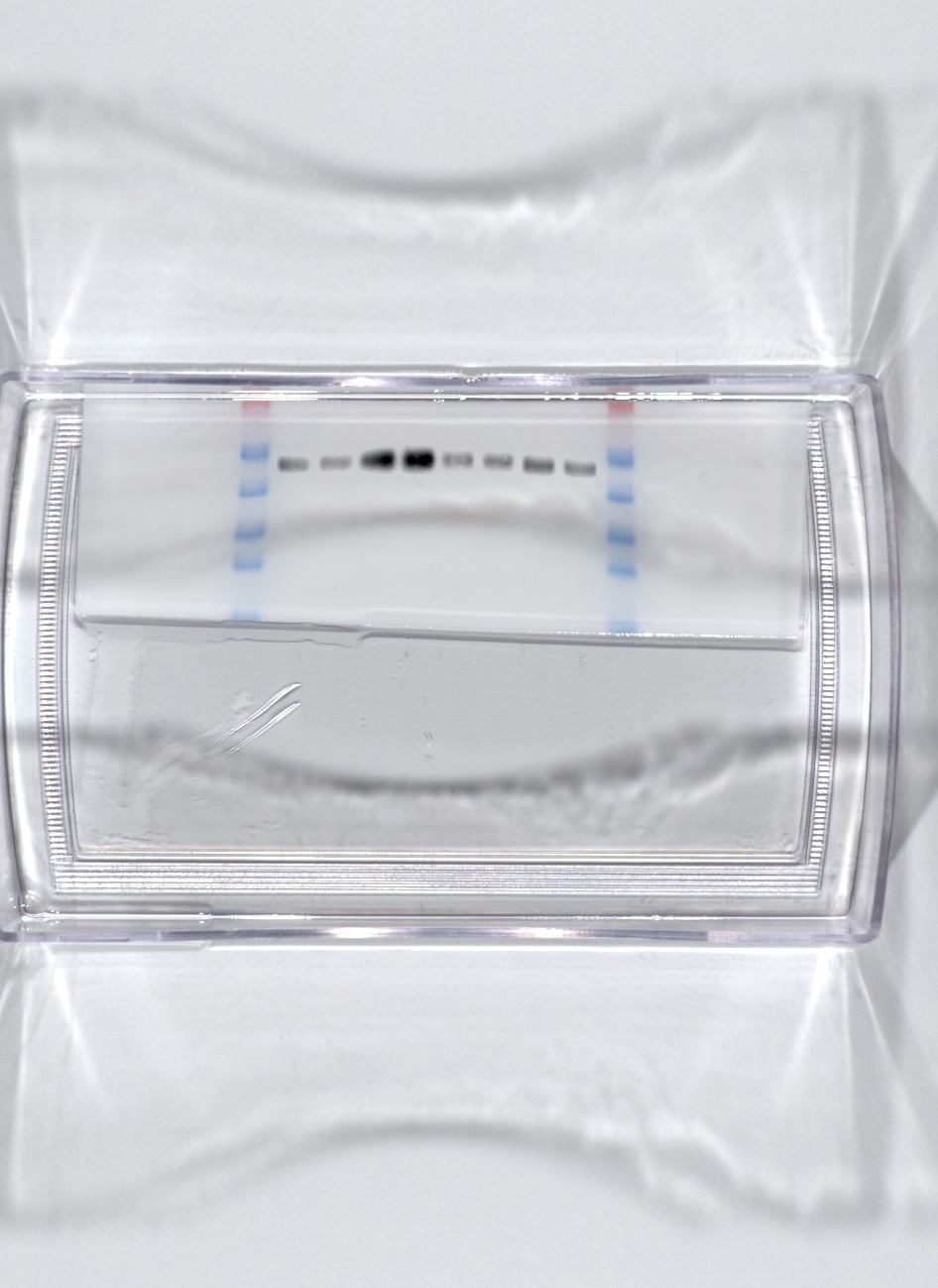
 α-SMA original blot


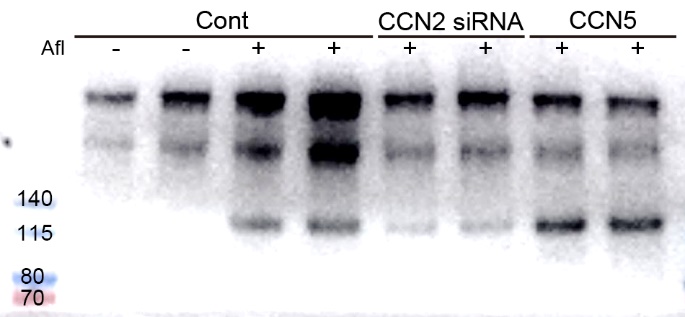


Fibronectin (250-kDa)


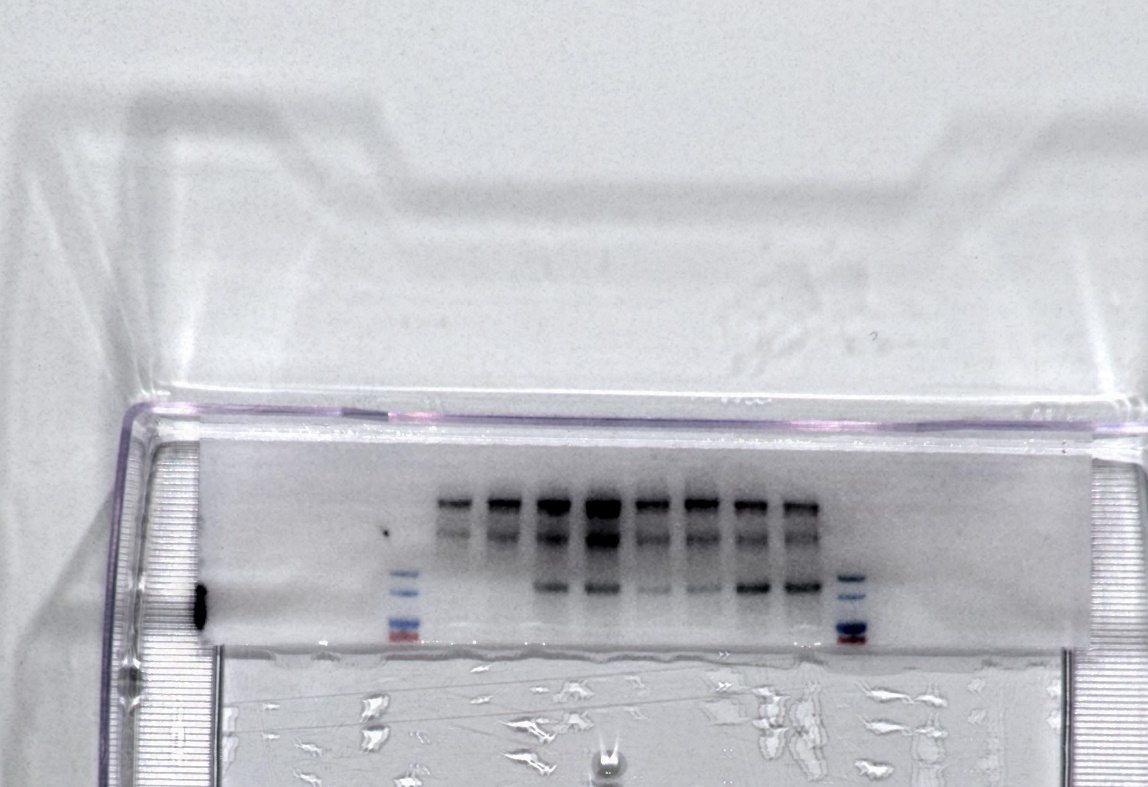
 Fibronectin original blot


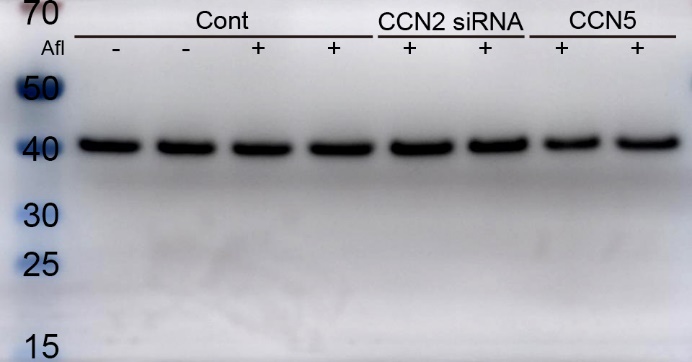


GAPDH (37-kDa)


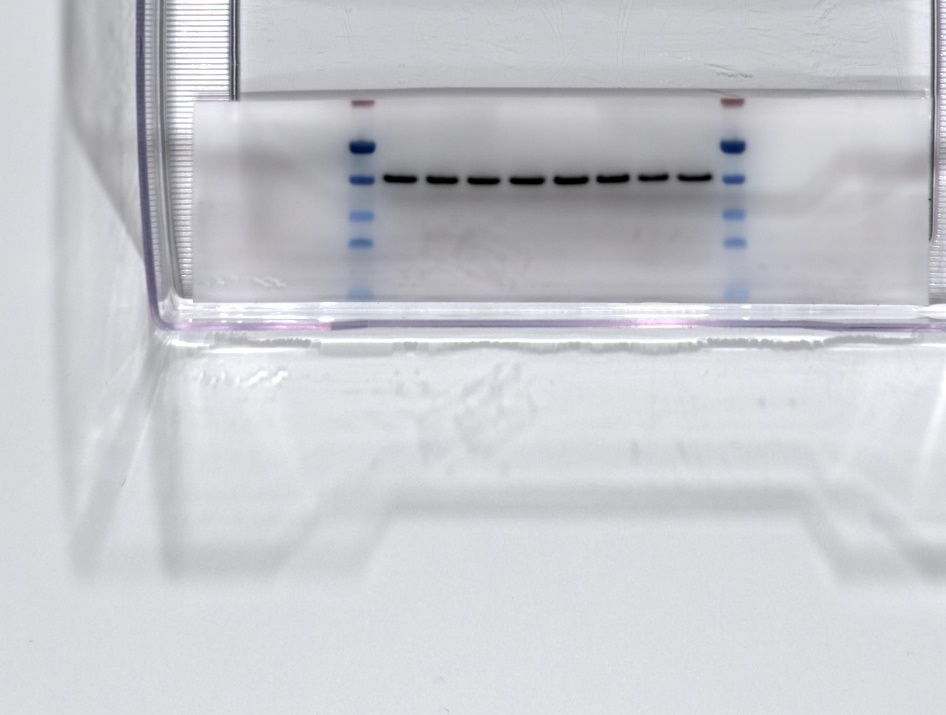
 GAPDH original blot

Supplementary Figure 1


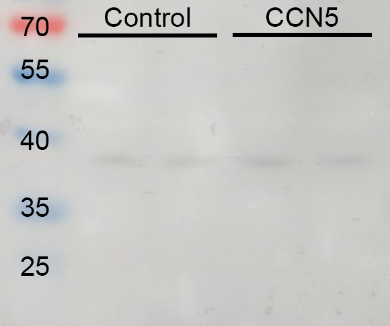


CCN2 (38-kDa)


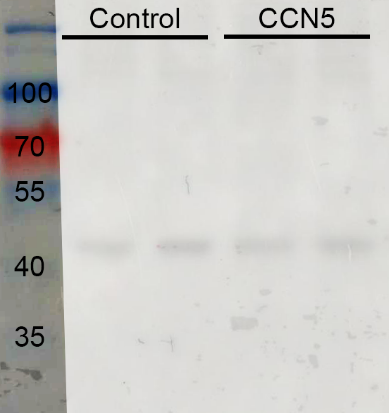


α-SMA (42-kDa)


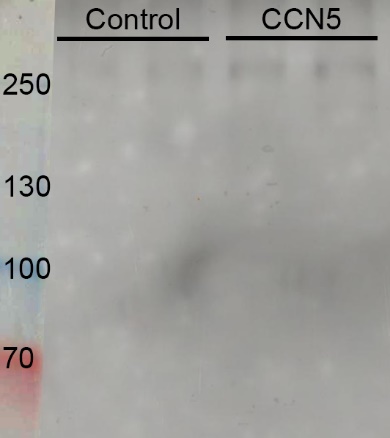


Fibronectin (250-kDa)


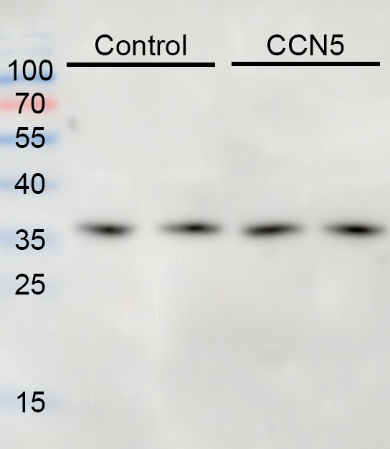


GAPDH (37-kDa)

Supplementary Figure 2


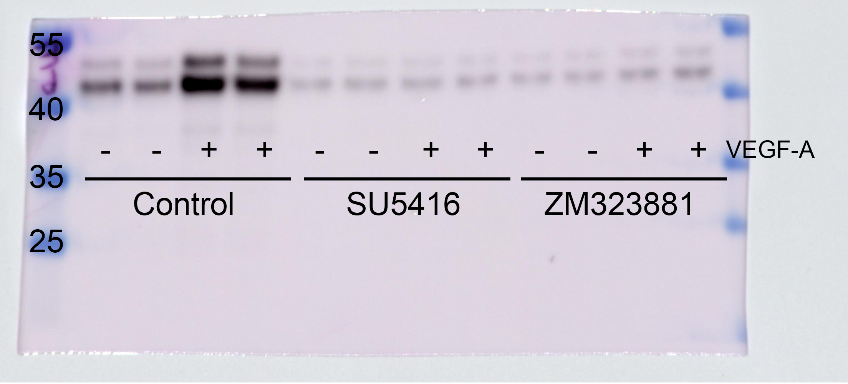


p-Erk1/2 (42, 44-kDa)


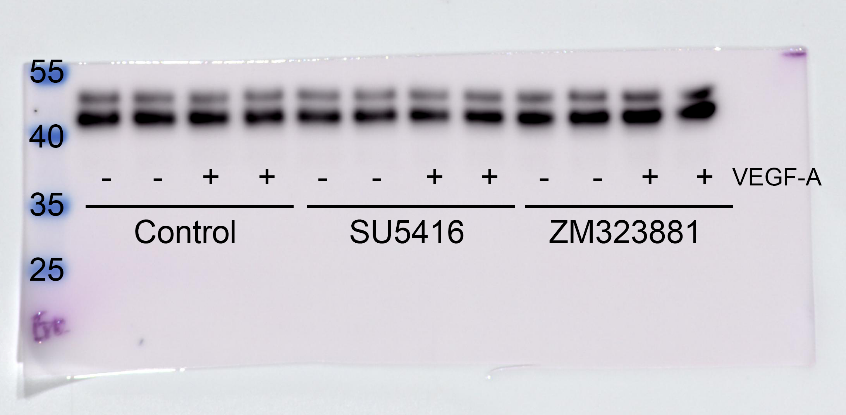


Erk1/2 (42, 44-kDa)


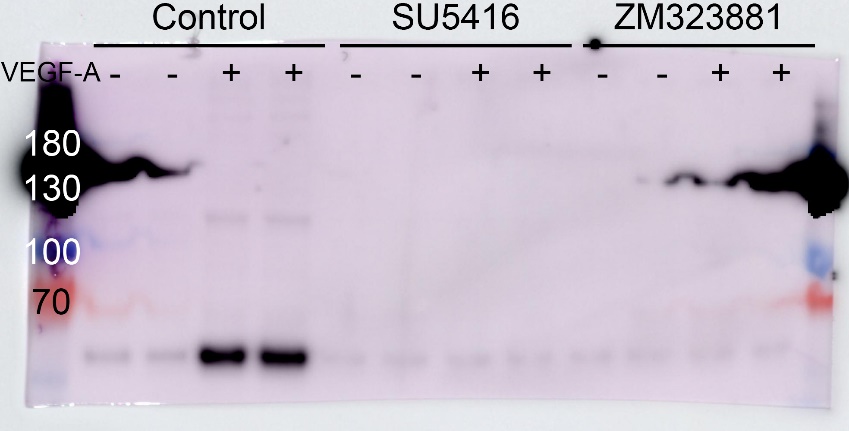


p-Akt (60-kDa)


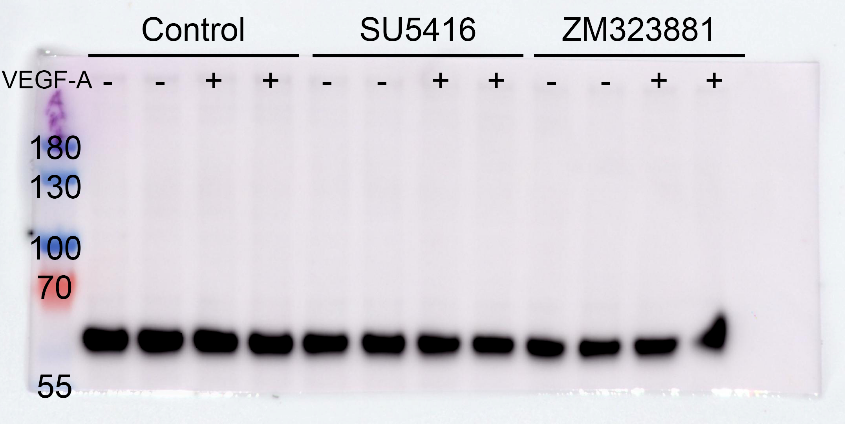


Akt (60-kDa)

Supplementary Figure 3


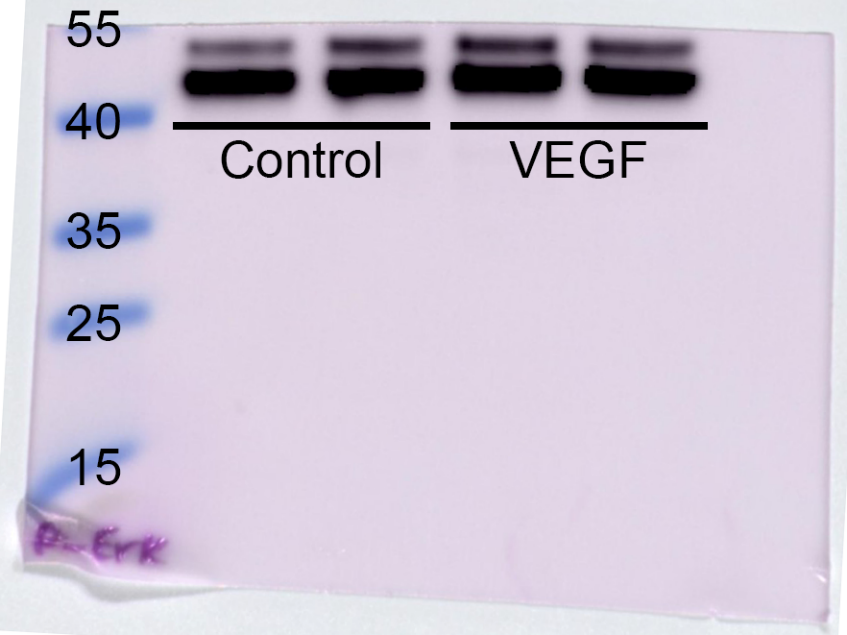


p-Erk1/2 (42, 44-kDa)


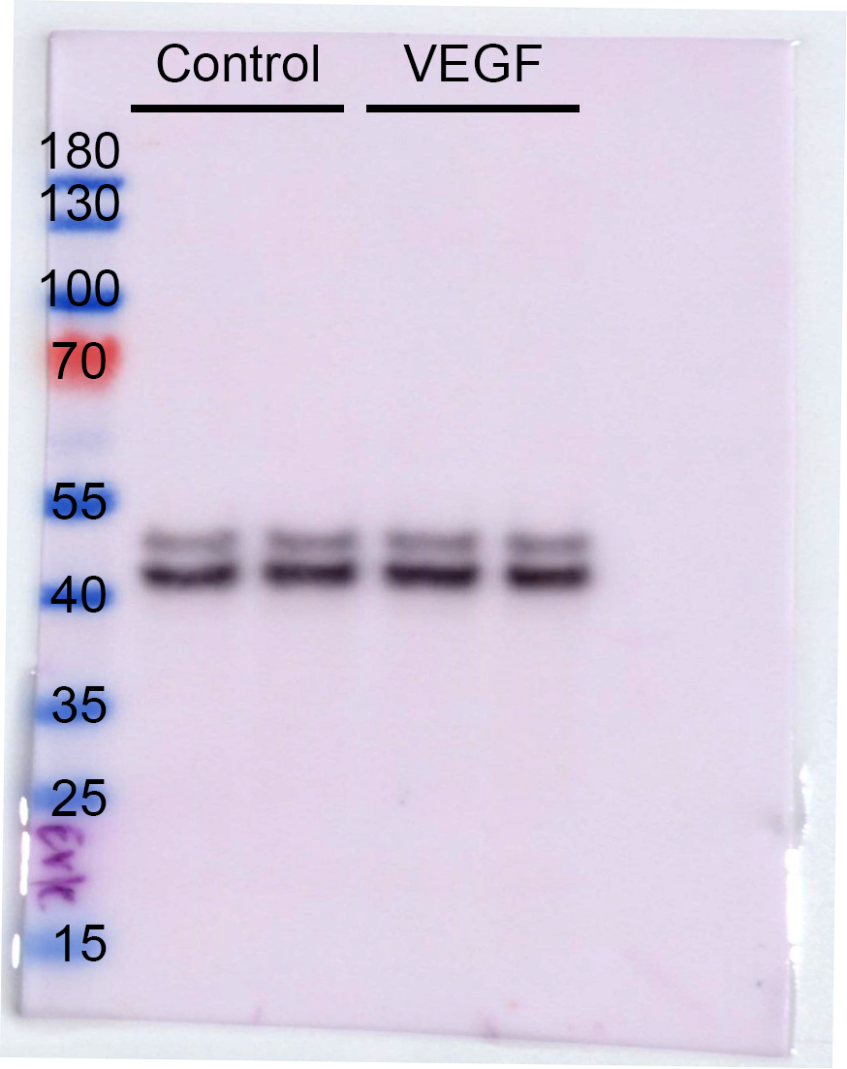


Erk1/2 (42, 44-kDa)


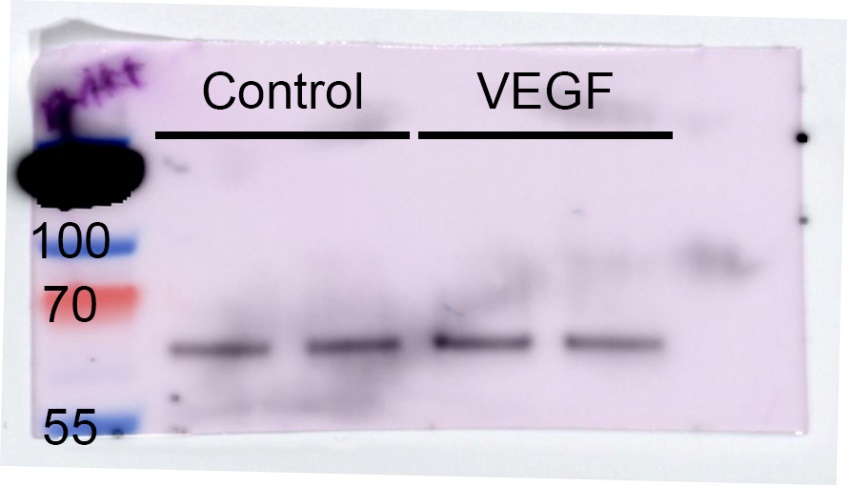


p-Akt (60-kDa)


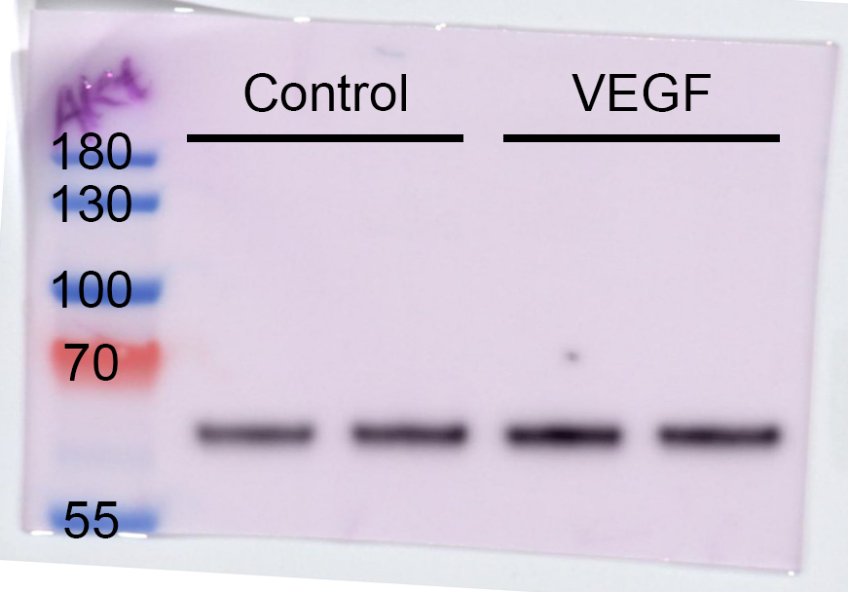


Akt (60-kDa)
