# Supplementary material for: The matricellular protein CCN5 prevents anti-VEGF drug-induced epithelial-mesenchymal transition of retinal pigment epithelium
Source: Sci Rep. 2024 Jun 17;14:13920. doi: 10.1038/s41598-024-63565-z (PMC11183261; doi:10.1038/s41598-024-63565-z)
Supplement: Supplementary file 2 — Supplementary Information. [file 41598_2024_63565_MOESM2_ESM.docx]

**Supplementary Figures**

**The matricellular protein CCN5 prevents anti-VEGF drug-induced epithelial-mesenchymal transition of retinal pigment epithelium**

Sora Im, Min Ho Song, Muthukumar Elangovan, Kee Min Woo, and Woo Jin Park

**Supplementary Figure 1.**


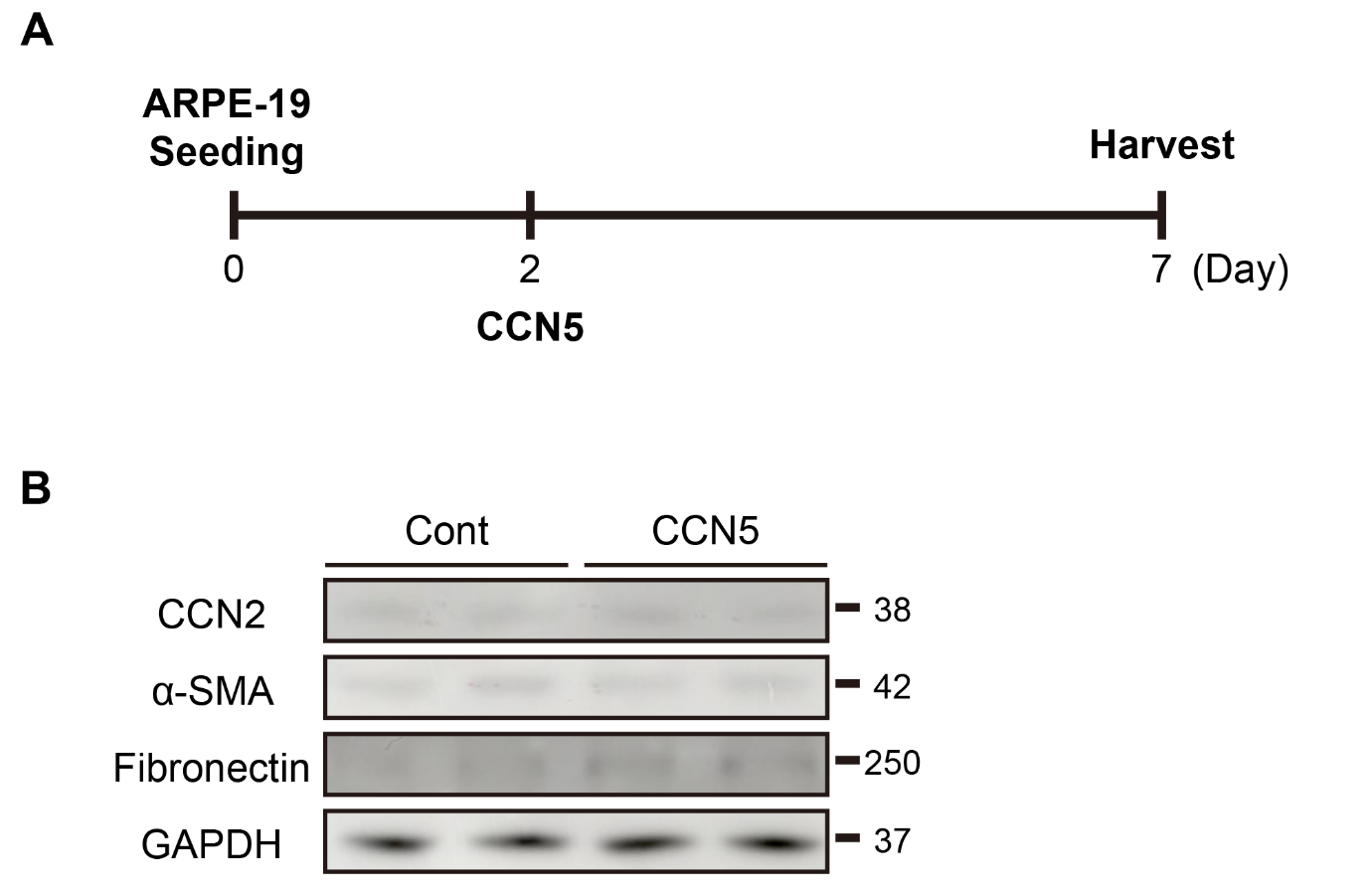


**Supplementary Figure 1.** CCN5 does not induce EMT in ARPE-19 cells. (A) An experimental scheme. The ARPE-19 cells were treated with recombinant CCN5 protein (500 ng/mL). (B) Cell lysates from ARPE-19 cells were immunoblotted with antibodies against CCN2, α-smooth muscle actin (α-SMA), fibronectin, and GAPDH.

**Supplementary Figure 2.**


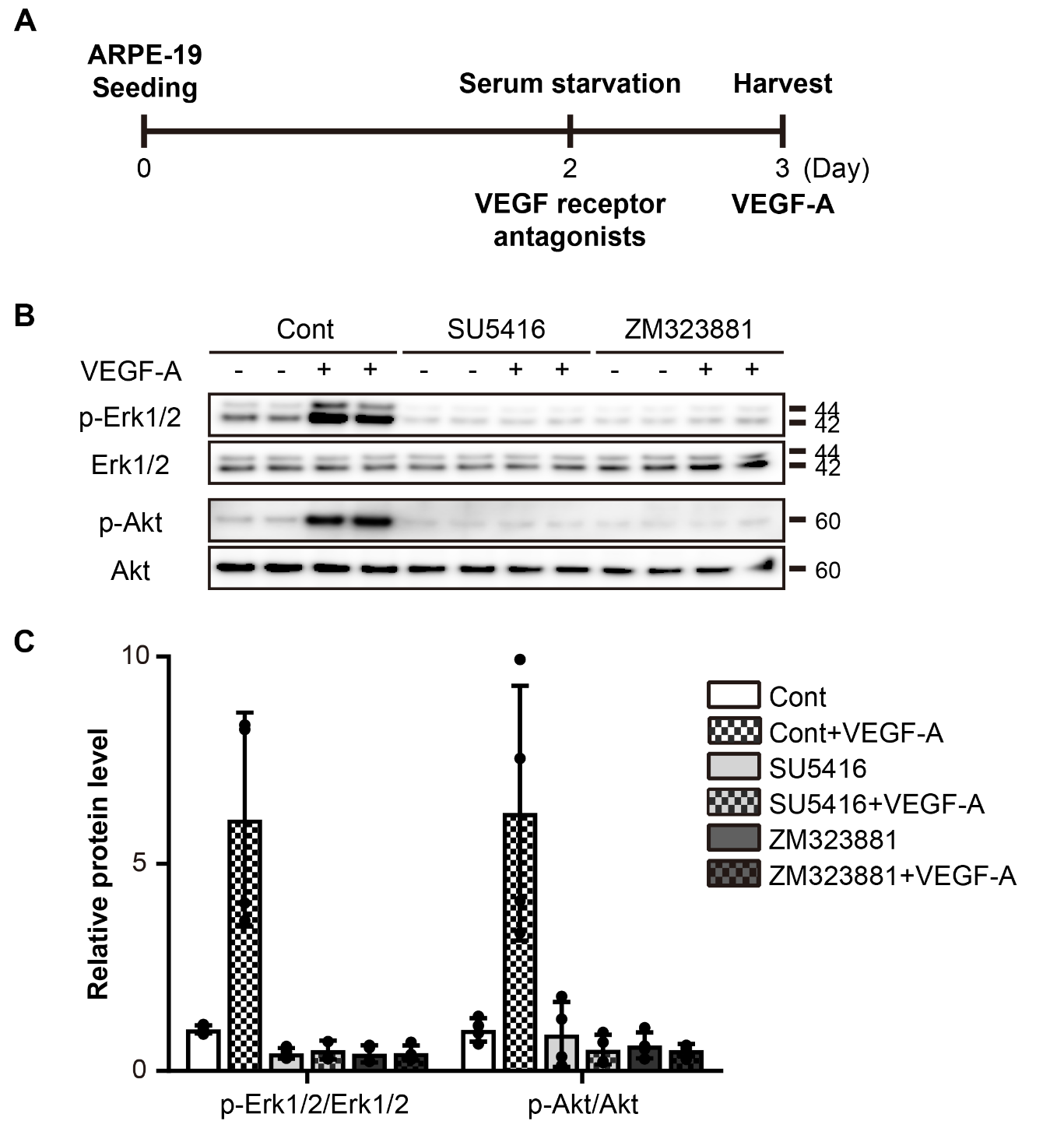


**Supplementary Figure 2.** Antagonists of VEGF receptor inhibit VEGF signaling pathway in ARPE-19 cells. (A) An experimental scheme. The ARPE-19 cells were treated with 5 μM of SU5416 or 10 nM of ZM323881 in serum free culture media. After 24 h treatment of antagonists, ARPE-19 cells were treated with VEGF-A (20 ng/mL) for 20 min. (B) Cell lysates from ARPE-19 cells were immunoblotted with antibodies against phospho-Erk1/2, Erk, phosphor-Akt, and Akt. (C) Protein levels were quantified using Image-J software and plotted. Bar show the mean ± SD. (n=4 per group)

**Supplementary Figure 3.**


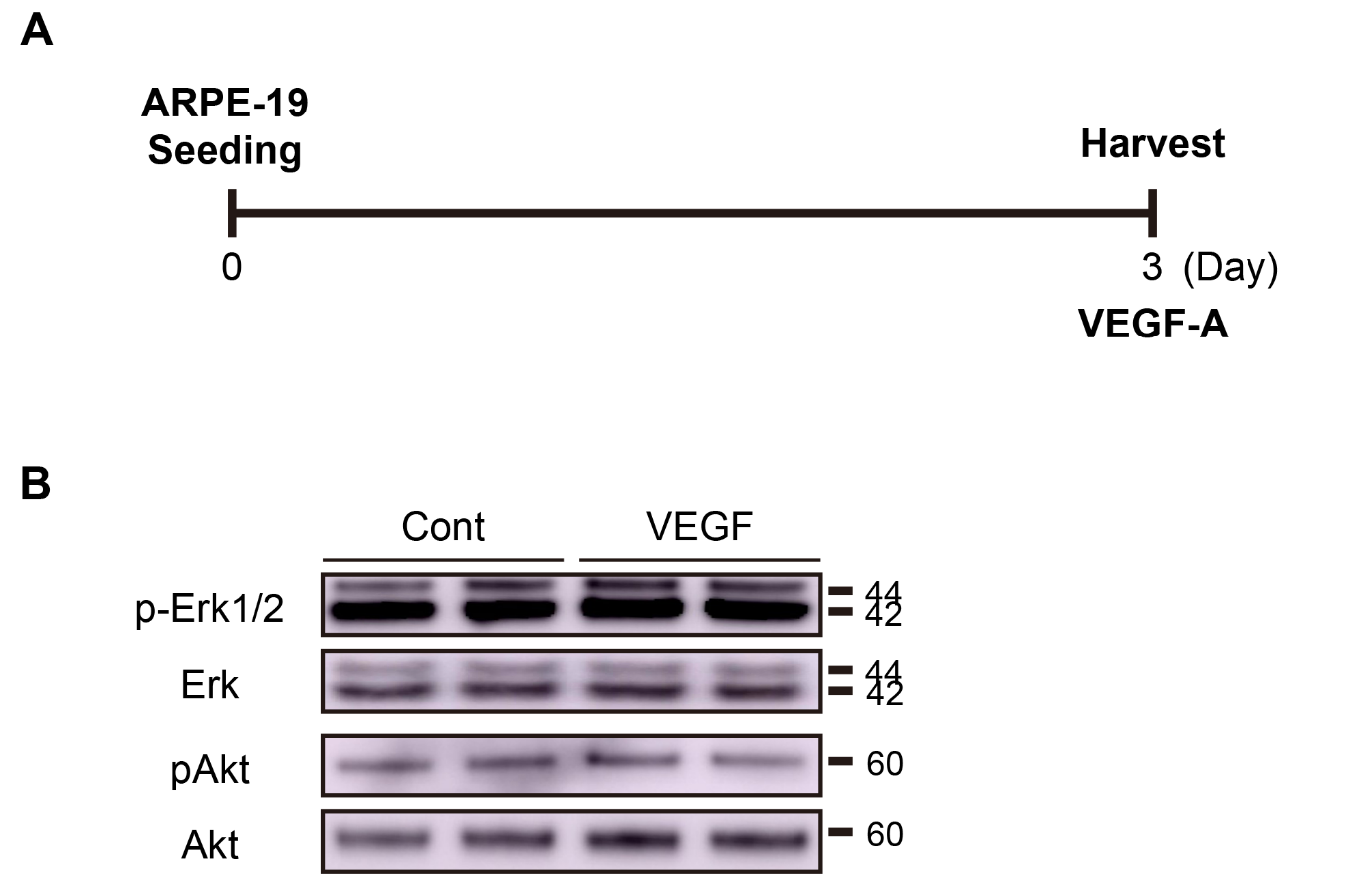


**Supplementary Figure 3.** Constitutive activation of VEGF signaling in ARPE-19 cells. (A) An experimental scheme. The ARPE-19 cells were treated with VEGF-A (20 ng/mL) for 20 min. (B) Cell lysates from ARPE-19 cells were immunoblotted with antibodies against phospho-Erk1/2, Erk, phosphor-Akt, and Akt.
